# A Basis for Strengthening Coping Strategies and Treatment Expectations in Patients Undergoing Emetogenic Pelvic-Abdominal Radiotherapy: A Longitudinal Study

**DOI:** 10.1177/15347354241281329

**Published:** 2024-09-20

**Authors:** Anna Efverman, Marja-Leena Kristofferzon

**Affiliations:** 1 University of Gävle, Gavle, Sweden

**Keywords:** body-mind, cancer care, coping, emesis, expectancy, integrative oncology, mental adjustment, nursing, nausea, oncology care

## Abstract

**Objectives:** To study the relationship between coping strategies and nausea during emetogenic pelvic-abdominal radiotherapy, and to describe the patients’ rationales for their expectations regarding nausea. **Methods:** Patients (n = 200: 84% women, mean age 64 years, 69% had gynecological, 27% colorectal, and 4% had other malignances) longitudinally participated during pelvic-abdominal radiotherapy. We measured adopted coping strategies using the Mental Adjustment to Cancer scale and the patients’ rationales for their expectations regarding nausea at baseline. The patients registered nausea and vomiting daily during the radiotherapy period (mean 36 + Standard Deviation 10 days). **Results:** Patients who experienced nausea (n = 128, 64%) during the radiotherapy period graded a lower score on “Fighting Spirit” (median, md, score 51, P = .031) and a higher score on “anxious preoccupation” (md 18, P = .040) compared to patients who did not experience nausea (n = 72, 36%), md 54 and md 17. More of the patients for whom “Helpless-Hopeless” represented the most predominant response experienced nausea (100%) or vomited (56%) compared to patients adopting “Fighting Spirit”: 62% experienced nausea (P = .011) and 20% vomited (P = .014). Only four (6%) of the nausea-free patients had expected themselves to be at increased risk for nausea. Of the patients who became nauseous, 22 (17%) patients had expected themselves to be at increased risk for nausea (P = .017), based on previous experience. **Conclusion**: Patients adopting maladaptive coping strategies or patients expecting nausea based on previous experiences, were more likely to experience nausea than other patients when undergoing emetogenic pelvic-abdominal radiotherapy. Cancer care professionals may identify patients adopting maladaptive coping strategies or having high nausea expectations by applying the MAC scale and self-assessment of expected nausea risk and guide these patients to adopt adaptive coping strategies and strengthen their expectations on successful prevention of nausea.

Trial registration number: Clinicaltrials.gov: NCT00621660.

## Introduction

Nausea is a stressing and demanding side-effect of pelvic-abdominal radiotherapy.^1-4^ Handling such demands requires coping strategies.^5,6^ Regarding non-cancer populations, women with emesis during pregnancy used a more helpless approach in their coping,^7^ while this association seems to not have been studied previously regarding patients undergoing emetogenic radiotherapy for cancer. Patients differ in the way they expect nausea during cancer therapy^8-10^ and expectations have the potential to modify the actual symptom occurrence.^9,11^ Integrative oncology is a patient-centered, evidence-informed field of cancer care that integrate therapies from different traditions, aimed to optimize health and to empower patients with cancer.^12^ Many patients report that integrative oncology may improve their coping strategies^13^ and relieve nausea.^14^ Integrative oncology would welcome more knowledge regarding coping strategies and expectations in relation to nausea in patients undergoing emetogenic radiotherapy.

Being diagnosed with a cancer in the pelvic-abdominal region and starting radiotherapy, with risk for side-effects,^1-4^ is highly demanding. Besides the impact of being diagnosed with a life-threatening disease, patients often also face psychosocial problems due to a variety of stressful and demanding side-effects.^2^ Nausea is ranked among the most incapacitating side-effects during cancer therapy,^1,10^ and is experienced by approximately 60% to 70% of the patients undergoing pelvic-abdominal radiotherapy.^1,3,4,8^ Radiotherapy involving abdominal or pelvic fields^1-4^ induces cellular damages in the gastrointestinal tract, which leads to release of serotonin. The serotonin activates serotonin-receptors on closely associated vagal afferent fibers, resulting in transmitter release in the vomiting center, causing the sensation of nausea. If the activity is strong enough, vomiting occurs. Afferents from the visual, vestibular, or limbic structures may also activate the vomiting center. Like learning effects in other contexts, the mechanisms regulating emetogenic stimulus act more effectively by an increased sensitivity through learning from previous expose for emetogenic stimulus, for example, experiences from chemotherapy- or pregnancy related nausea. Thus, biological mechanisms explain why stressful emotions or nausea expectations, learned by previous nausea experience, may induce nausea.^15^

Because of the demanding stressors, patients undergoing pelvic-abdominal radiotherapy risk decreased quality of life (QoL).^2,4,16^ Presence of nausea was seen to worsen QoL during pelvic-abdominal radiotherapy.^4^ A patient experiencing stress and demands naturally begins a cognitive and behavioral adaptive process to deal with the stressors and demands.^5,6^ Chemotherapy-induced toxicities have been more studied than radiotherapy-induced ones, and according to those studies, the stage of cancer and burden of the toxicity of the cancer therapy have been seen to affect QoL, functioning and symptom experience. The patient’s coping strategies for responding to a stressor or a demand altered these outcomes.^6,17-19^

Roy’s Adaptation Model^20,21^ describes the patient as a biopsychosocial individual, constantly interacting with, and adjusting to the surrounding environment. Patients thus apply a variety of required mechanisms to adapt and adjust to the situation. Two subsystems of the adjustment process are described. The “regulator subsystem” is the more basic type of adaptive process. This subsystem responds automatically through neural, chemical, and endocrine adapting channels,^21^ involving activation of the vomiting center in patients undergoing radiotherapy.^15^ The “cognator subsystem” is described as being a process involving the cognitive-emotive channels; perceptual and information processing; learning from previous experiences, judgments or expectations; and emotions. The proposal for two subsystems affecting each other provides the theoretical basis for why different coping strategies potentially, though not previously known, give different outcomes, for example, regarding nausea. In patients with cancer specifically, coping may be defined as the cognitive and behavioral responses the patient makes to the diagnosis of cancer, cancer therapies, and cancer related symptoms. The adapting process comprises all kinds of cognitive and behavioral responses undertaken by a person in a situation of stress and demands related to the cancer.^5^ Coping strategies of managing stressing and demanding situations seem to depend on individual factors; a set of strategies for overcoming stress seem to be characteristic for a given person.^22^ However, clinical, and contextual factors, such as support, may modify the choice of coping strategy that will dominate in the given situation,^23-26^ giving a basis for potential effects of integrative therapies.^13,14^ The response to and management of the stressor may be a positive or a negative active solution, or no solution at all, just being passive.^22^ Patients have experienced that integrative oncology, adopting a holistic biopsychosocial perspective, improved their coping strategies.^13^ Since support in the contextual situation may modify the choice of coping strategy that will dominate in the given situation,^13,23^ it seems plausible that cancer care professionals’ support during demanding radiotherapy may be of importance for the choice of coping strategy.

Patients who confronted demands felt a higher level of optimism, appeasement, and self-reliance and experienced better QoL during radiotherapy for cervical cancer,^25^ while the association with nausea seems not to have been studied yet. “Fighting Spirit” responses have been described as a highly optimistic attitude, accompanied by an active search for information about the situation.^5^ “Fighting spirit” was related to a better QoL^24^ and psychological health.^19,24^ Other coping strategies, such as “Helpless-Hopeless” responses^27^ indicate loss of hope and visualizing oneself as being gravely ill.^5^ Such responses were related to poorer QoL and psychological health.^6,17^ Maladaptive coping strategies such as “Anxious preoccupation” and “Helpless-Hopeless” indicated a greater risk for impairing chemotherapy-induced nausea for those employing such strategies than for those who were able to employ “Fighting spirit” responses.^28^

Interviewed patients expressed that their expectations, based on previous experiences, were highly important for their nausea experiences during chemotherapy.^10^ Patients who see stimuli as rational and explainable, that is, in line with what they expected, have been shown to have a high capacity to cope with stress and be more likely than others to maintain QoL during cancer illness.^6,17^ Previous studies during chemotherapy regarding the role of expectations on nausea for the actual nausea occurrence indicate the need for further studying this relationship^9,10^ also during radiotherapy. Patients during radiotherapy expect support from cancer care professionals regarding strategies that sufficiently manage stressors and demands.^29^ Thus, to be able to meet unmet patient needs during emetogenic radiotherapy, cancer care professionals in integrative oncology need more knowledge regarding coping strategies and expectations in relation to nausea.

The objective of the current paper was to study the relationship between coping strategies and nausea during emetogenic pelvic-abdominal radiotherapy, and to describe the patients’ rationales for their expectations regarding nausea.

## Method

### Design

This paper adopted a longitudinal descriptive design, using the same data set as a previously reported randomized sham-controlled study regarding the effect of antiemetic acupuncture compared to sham acupuncture.^8^ Because there were no differences between the randomization groups regarding nausea, vomiting, coping, expectations, or any other outcomes, we included all patients in the current study. The study was registered, followed the ethical standards of the Helsinki declaration, and had ethical approval (approval number 02-420, date 2002-11-05, and M167-04, date 2004-12-14).

### Patients and Setting

Study coordinating nurses at each study site provided written study information to all patients scheduled for fractionated external pelvic-abdominal radiotherapy at 2 Swedish University Hospitals, and informed them that they were going to receive oral study information from a study physiotherapist. The study physiotherapist briefly summarized the written study information during a telephone call and screened the patients for study criteria and willingness to participate. A consecutive sampling procedure was applied. Inclusion criteria: Patients who were at least 18 years of age and who had gynecologic, anal, rectal, colon, stomach, pancreatic or testicular cancer were included if they were planned for radiotherapy to an abdominal or pelvic field of at least 800 cm^3^ volume and 25 Gray dose, and if they had the physical, mental, and linguistic capacity to give informed consent. Only patients delivering at least 1 week of nausea measures were included in this paper. Exclusion criteria: Antiemetic treatment or persistent nausea or vomiting within 24 hours before start of radiotherapy, previous acupuncture during the year preceding the inclusion regardless of the reason for being given acupuncture, or previous acupuncture given specifically for nausea or vomiting at any previous time preceding the inclusion.

**Figure 1.**
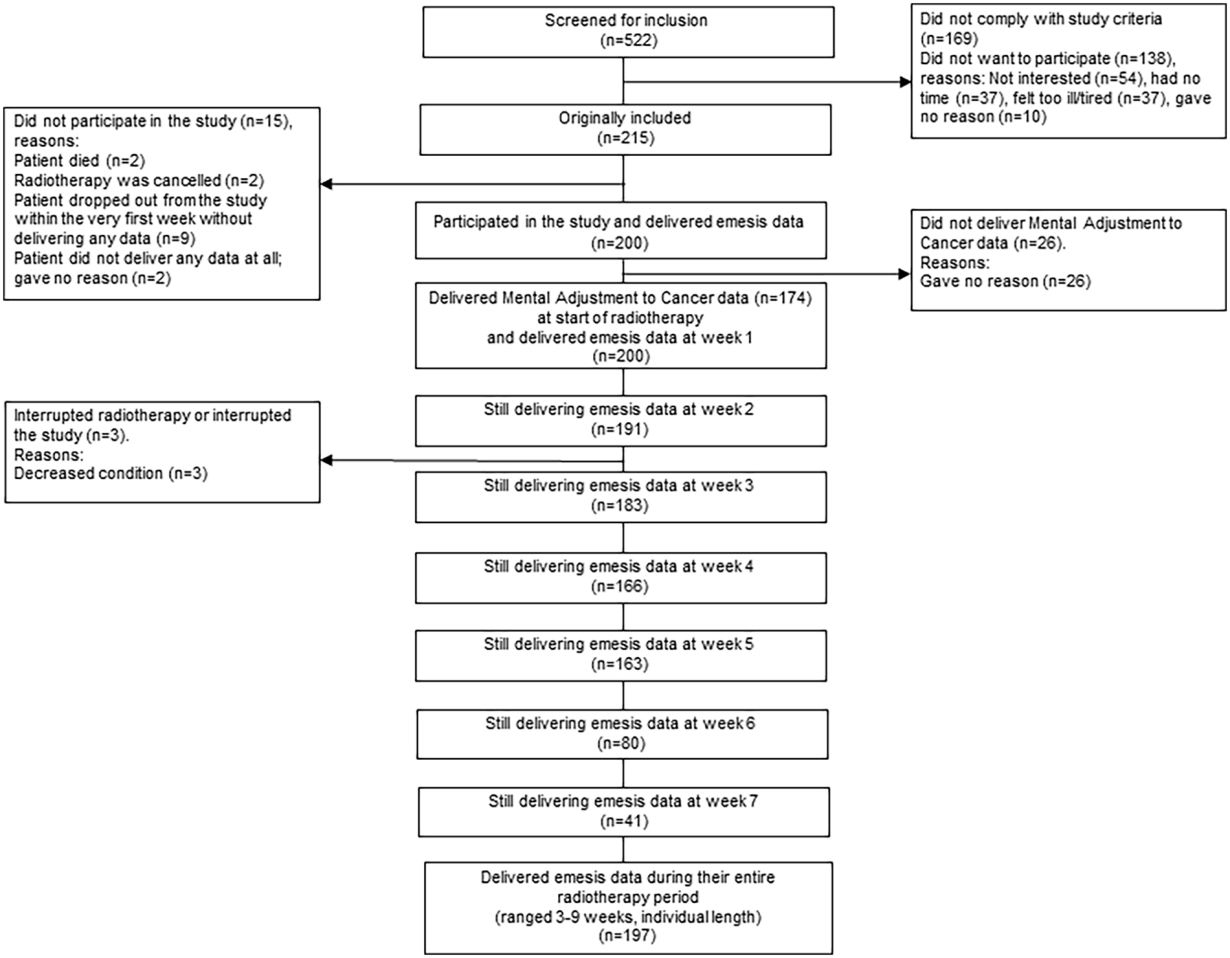
Numbers of patients screened, included and delivering data when undergoing radiotherapy.

Of the 522 consecutively screened patients during a 3-year period, 169 did not meet the study criteria (eg, not receiving radiotherapy to pelvic-abdominal regions), 138 did not want to participate, while 215 were possible to include in the current study. Of these patients, 15 (7%) did not deliver at least 1 week of nausea data (2 patients died, radiotherapy was cancelled for 2 patients, 9 patients dropped out from the study within the first week before they delivered the nausea registrations, and two patients did not deliver any data at all for unknown reasons). Accordingly, 200 patients participated in the current study (Figure 1).

The patients received radiotherapy for a median time of 5 weeks, 1 fraction a day, mostly as outpatients. Oncologists prescribed rescue pharmacological treatment for nausea^3^ according to ordinary clinical routines specified for various dose levels in The Swedish Medicine Information Engine (http://www.fass.se). All patients also received non-pharmacological antiemetic treatment, covering acupuncture using penetrating or non-penetrating needles, 2 to 3 times a week during the entire radiotherapy period. These standardized treatment routines have previously been described.^8^

### Data Collection

The study-coordinating nurse at each study site collected clinical descriptive data from the patients’ medical record. The patients responded to written descriptive questions on sociodemographic characteristics the day before the start of radiotherapy. The study-coordinating nurse also delivered the longitudinally applied questionnaires as described below. The patients answered them in writing (analog, using pen and paper), and posted them to the study evaluator in response boxes at the hospital or in regular postal mailboxes, using pre-paid response envelopes. The patients were informed that neither the study coordinator nor the cancer care professionals would read their responses. If the patients had not delivered any responses approximately 1 week after responses to each questionnaire were expected, the evaluator notified the study-coordinating nurse to make one reminder (personally if the patient was still undergoing radiotherapy, or by phone if the radiotherapy was completed). All data were confidentially handled, by using a code number for identification instead of using any personal information.

#### Assessment of coping strategies

The day before their first fraction of radiotherapy, the patients provided written answers regarding their coping strategies with the widely used^6,17,26-28^ Mental Adjustment Cancer (MAC) scale,^5^ which may be applied irrespective of cancer type, cancer therapies, or symptoms. The scale assesses coping strategies in relation to cancer related stress and demands in general.^5^ The MAC scale has been translated to Swedish,^27^ with satisfactory validity and reliability.^27^ The patients assessed each of the MAC scale’s 40 items on a four-graded category scale: “totally disagree,” “partly disagree,” “partly agree” or “totally agree.” The items result in 5 domains, according to the factor-analysis for the original version^5^ as well as the Swedish version used in the study^27^: Fighting Spirit (16 items, the domain score ranges 16-64), for example, “I try to fight the illness,” Helpless-Hopeless (6 items, score ranges 6-24), for example, “I feel completely at a loss about what to do,” Avoidance (1 item, score ranges 1-4), that is, “I distract myself when thoughts about my illness come into my head,” Anxious Preoccupation (9 items, score ranges 9-32), for example, “I worry about the cancer returning or getting worse,” and Fatalism (8 items, score ranges 8-32), for example, “I’ve had a good life; what’s left is a bonus.” A higher score on a domain means that the responding patient adopts that kind of coping strategy to a higher extent than a patient with a lower score.

#### Assessment of patients’ rationales for their expectations on nausea

The day before the first radiotherapy fraction, the study questionnaire^8^ assessed expectations on nausea by asking the patients to estimate their own risk for nausea: “In relation to others, how do you estimate your own risk for becoming nauseous during the radiotherapy period?” on a five-graded category scale by choosing one of the response alternatives “Much lower risk,” “Lower risk,” “Similar risk”, “Higher risk,” and “Much higher risk.” The patients, using their own words, expressed in writing their rationales for their estimation.

#### Assessment of nausea and vomiting

During the entire radiotherapy period (median 5 weeks) the patients daily and in privacy (ie, at home/ward unit/patient hotel) responded to a valid and reliable emesis-questionnaire.^4^ The questions analyzed in this study were: “Have you within the past 24 hours experienced nausea?” (“No” or “Yes”), and “Have you within the past 24 hours vomited?” (“No” or “Yes”).

### Statistical Analyses

The independent evaluator (ie, not involved in the data collection) summed the scores of items within the 5 MAC-scale domains. The patients were categorized, based on all the daily longitudinally answered emesis-questionnaires, into 2 groups, one “Experienced nausea” (patients experiencing nausea at least one episode) and the other, “Not experiencing nausea” (patients who were free from nausea during the entire radiotherapy period), and we followed the same procedure regarding vomiting. Then, the 5 MAC-scale domain scores [medians (md), 25th-75th percentiles] for patients experiencing and not experiencing nausea were compared and the same was done for patients experiencing and not experiencing vomiting, using the Mann-Whitney *U*-test. The evaluator also calculated number (n) and percent (%) of patients for whom each of the MAC-scale domains represented the most predominant response (domain reaching the highest summed score divided by the number of domain items) and compared the patients with different dominant strategies regarding occurrence of nausea and occurrence of vomiting using the chi-square test, presented as Relative Risks (RR) for nausea, and for vomiting, with 95 percent confidence intervals (CI).

The number and percentage of patients expecting themselves to be at different levels of risk for nausea, and the number of patients expressing different rationales for their expectations, was calculated. The Chi-square test compared patients who expected themselves to be at higher risk (greater or much greater risk) or not (similar, lower, or much lower risk in relation to others) regarding nausea experience during the radiotherapy period, presented as RR with CI. The evaluator classified similar rationales into larger categories. For example, the answers “Since I used to experience nausea” and “Nausea often bothers me,” were both assigned to the single category “Often experience nausea in general.”

The analyses were performed in IBM SPSS for Windows (Armonk, NY: IBM Corp), version 24.0. The significance level was set at 5%, using 2-sided tests.

## Results

### The Patients

Table 1 presents the demographics of the patients. Most of the 200 patients (mean age 64 years) were women receiving radiotherapy for a gynecological malignancy (84% were women, 69% had a gynecological malignancy). Twenty-six of the patients did not deliver MAC-scale data while 174 patients responded to the MAC scale. Of the 174 patients, 42 patients failed to respond to at least one item, while 132 patients (92% women, mean age 61 years, 68% had a gynecological malignancy) answered all 40 items.

**Table 1.** Demographics of the Patients.

Variable	Total study group, n = 200
Sex, n (%)	
Man	32 (16)
Woman	168 (84)
Age in years, m ± SD	63.9 ± 13.5
Marital status, n (%)	n = 192
Married or living together	120 (62)
Living alone, have a partner	12 (6)
Living alone, have no partner	60 (31)
Employment status, n (%)	n = 199
Employed	75 (38)
Student	2 (1)
Not employed (Housewife/husband)	4 (2)
Retired/Sickness pension	118 (59)
Medication for any other illness than cancer, n (%)	n = 199
Yes	168 (84)
No	31 (16)
Cancer type, n (%)	
Gynecological	138 (69)
Colon, rectal, anal	54 (27)
Testicular	2 (1)
Pancreas, ventricular	6 (3)
Clinical status, n (%)	n = 199
Treated as out-patient	172 (86)
Treated as in-patient	27 (14)
Irradiated field, n (%)	
Abdomen	13 (7)
Pelvis	187 (94)
Radiated dose (Gy) m ± SD	49.1 ± 10.6
Radiated volume (cm3), m ± SD	1424 ± 608
Length of radiotherapy period in days, m ± SD Range	36 ± 10 2-69
Received concomitant chemotherapy, n (%)	n = 199 57 (29)
Abdominal surgery last 6 mo, n (%), md, 25th-75th percentile number of weeks since surgery	n = 184 80 (43), 12, 8-21

N and proportions (%) of patients answering the questions are presented.

Abbreviations: n, number; m, mean; ±SD, standard deviation; md, median; Gy, Gray; cm, centimeter.

### Coping Strategies in Patients With Different Nausea and Vomiting Experiences

**Figure 2.**
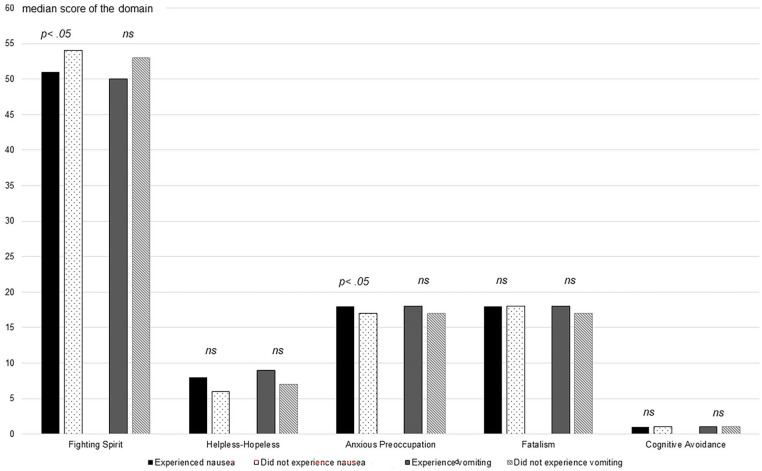
The variety of coping strategies presented as median scores of the Mental Adjustment to Cancer scale domains in patients experiencing or not experiencing nausea and vomiting during radiotherapy. ns, not a statistically significant difference; the significance level was set at 5%.

Of the 200 patients, 128 (64%) had experienced at least 1 episode of nausea while 72 (36%) were free from nausea during the entire radiotherapy period. Patients who did not experience nausea during the radiotherapy period reported a higher score on the domain “Fighting Spirit” (P = .031) and a lower score on the coping strategy “Anxious Preoccupation” (P = .040) compared to the patients experiencing nausea during the radiotherapy period. The tendency for the occurrence of higher Helpless-Hopeless scores in responses by the vomiting patients compared to patients free from vomiting did not reach statistical significance (P = .065; Figure 2).

All 10 of the patients for whom “Helpless-Hopeless” represented the most predominant response experienced nausea (100%) and 5 reported vomiting (56%) during the radiotherapy period. For patients for whom “Fighting Spirit” represented the most predominant response, the percentages were lower, with 55 (62%, P = .011) experiencing nausea and 17 (20%, P = .014) experiencing vomiting (Table 2).

**Table 2.** Coping Strategies for Mental Adjustment in Patients Experiencing or Not Experiencing Nausea During Radiotherapy.

MAC-scale domain Median (25th-75th percentile); n (%) of patients for whom each of the domains represents the most predominant response	Experienced nausea n = 128	Did not experience nausea n = 72	Relative risk for experiencing nausea 95% CI	Experienced vomiting n = 53	Did not experience vomiting n = 147	Relative risk for experiencing vomiting 95% CI
Fighting sprit	51 (50-56) 55 (62)	54 (49-56) 34 (38)	Ref	50 (42-55) 17 (20)	53 (49-56) 70 (81)	Ref
Helpless/Hopeless	8 (6-11) 10 (100)	6 (6-9) 0 (0)	1.6 (1.4-1.9)^a^	9 (6-13) 5 (56)	7 (6-9) 4 (44)	2.8 (1.4-5.9)^a^
Anxious preoccupation	18 (16-22) 6 (75)	17 (14-20) 2 (25)	1.2 (0.8-1.9)	17 (16-22) 2 (25)	18 14-21 6 (75)	1.3 0.4-4.6
Fatalism	18 (15-21) 5 (71)	18 (15-21) 2 (29)	1.5 (0.3-7.3)	18 (15-21) 2 (28)	17 (14-21) 5 (71)	1.5 (0.4-5.1)
Denial	1 (1-2) 7 (39)	1 (1-3) 11 (61)	0.6 (0.3-1.1)	1 (1-3) 5 (28)	1 (1-2) 13 (72))	1.4 (0.6-3.4)
Not classified	45 (58)	23 (42)	0.9 (0.8-1.3)	22 (31)	49 (69)	1.59 (0.9-2.8)

Median scores and interquartile ranges for each domain of the MAC scale are presented, and n and proportion (%) of patients for whom each of the domains represent the most predominant response.

Abbreviations: n, number; MAC scale, Mental Adjustment to Cancer scale; CI, confidence interval; Ref, Reference category, i.e., the coping strategy with the lowest proportion of nauseated/vomiting patients.

a Statistically significantly increased risk for nausea/vomiting. Of 200 study patients, 174 patients responded to MAC scale; 132 of these answered all 40 items and were classified to dominant domain, while 68 could not be classified due to internal lack of data ≥ one item. For the vomiting analyses, additional 3 patients could not be classified due to vomiting data were provided for less than 1 week.

### Patients’ Rationales for Expecting Themselves to Become Nauseous or Not

Of the 72 patients who did not experience nausea anytime during the entire radiotherapy period, only 4 patients (6%) had expected themselves to be at increased risk for nausea. Of the 126 patients who experienced nausea during the radiotherapy period, 22 (17%) had expected themselves to be at increased risk for nausea (RR 3.14, CI 1.13-8.76, P = .017). Rationales for this were that they had previous experiences regarding nausea. Also, although less frequently expressed, some patients believed they were at higher risk based on the body-location of the radiotherapy field, because they had heard about nausea from the oncologist and others, or because they had physical limitations that they believed implied an increased risk for nausea. Of the patients who expressed rationales for expecting themselves to be at lower risk for nausea and did not become nauseous, common rationales were that they had not experienced nausea in other situations and, although more rarely expressed, their belief in destiny/chance, and that they believed a positive personality to be beneficial (Table 3).

**Table 3.** The Patients’ Rationales for Expecting Themselves to Be at Different Levels of Risk for Nausea When Undergoing Radiotherapy.

Rationales for expected risk for nausea	Total study group n = 198^a^	Free from nausea n = 72	Experienced nausea n = 126^a^
Rationales for believing being at *much higher* risk than others	4	2	2
Experience nausea when consuming medications	1		1
Experienced nausea during chemotherapy			
Gave no rationale; just rated risk in this way	3	2	1
Rationales for believing being at *higher* risk than others	22	2	20
Often experience nausea in general	1		1
Experienced nausea during chemotherapy	5	1	4
Experience motion illness	5		5
Experienced post-operative nausea	2		2
Experience nausea when consuming medications	1		1
Due to physical limitations^b^	1		1
Based on the body-location of radiotherapy, abdomen	1		1
Based on information from the oncologist	1		1
Gave no rationale; just rated risk in this way	5	1	4
Rationales for believing being at *similar* risk than others	111	38	73
Seldom experience nausea in general	4	1	3
Experienced nausea during chemotherapy	2		2
Experienced post-operative nausea	1		1
Due to physical limitations^b^	1		1
Due to psychological stability	1	1	
Due to physical stability	1	1	
Have heard from others regarding nausea	1		1
Have not heard anything from others regarding nausea	1		1
Experience heartburn	1		1
Based on information from the oncologist		1	
Seems individual or based on chance	6	3	3
Gave no rationale; just rated risk in this way	91	31	60
Rationales for believing being at *lower* risk than others	36	16	20
Seldom experience nausea in general	14	4	10
Did not experience nausea during chemotherapy	1		1
Experience motion illness	1	1	
Are capable to relax	1		1
Due to physical limitations^b^	2		2
Due to optimistic personality	1	1	
Seems individual or based on chance	1	1	
Gave no rationale; just rated risk in this way	15	9	6
Rationales for believing being at *much lower* risk than others	25	14	11
Seldom experience nausea in general	4	4	
Did not experience nausea during previous chemotherapy	1	1	
Due to optimistic personality	1		1
Gave no rationale; just rated risk in this way	19	9	10

Categorized free-text responses on baseline rationales in patients self-rating expectations to be at different levels of risk for nausea when undergoing radiotherapy are presented. The 5-graded ordinal category scale ranged from “Much lower risk than others,” to “Much higher risk than others” to experience nausea during the radiotherapy period.

a The total number of patients self-rating their nausea risk was n = 198, while n = 2 patients (both become nauseous during the radiotherapy period) did not respond, reason unknown.

b The patients made 2 examples of physical limitations: blindness or deafness.

## Discussion

This study found that patients adopting the maladaptive coping strategies “Anxious Preoccupation” and “Helpless-Hopeless,” or who based on previous nausea experiences expected nausea, were more likely than other patients to experience nausea during pelvic-abdominal radiotherapy. Patients who did not experience nausea during radiotherapy employed the coping strategy “Fighting spirit” to a greater extent and displayed to a lower extent maladaptive coping strategies “Anxious Preoccupation” and “Helpless-hopeless” than did patients who experienced nausea. The patients’ reasons for expecting nausea during the radiotherapy period were mostly that they had previous nausea experiences.

The findings indicate the importance of considering the link between body and mind for the nausea experience^11,30^ in patients undergoing emetogenic radiotherapy, which is in line with proposed biopsychosocial mechanisms of nausea,^15^ Roy’s Adaptation Model,^20,21^ and the fundamentals of integrative oncogy.^12^ This highlights the value of viewing the patient in a holistic biopsychosocial perspective. When discussing our findings in relation to previous research,^29,31-41^ most studies regarding this area are conducted during chemotherapy, whereas radiotherapy is much less researched. The findings regarding the relationship between type of coping strategies and nausea experience are in line with a previous study in chemotherapy care (n = 302).^28^ The patients who experienced nausea and vomiting reported higher scores on the domain “Helpless-Hopeless” compared to patients who did not experience these symptoms. The same relation was seen regarding “Anxious Preoccupation,”^28^ in line with the present study. Approximately a fourth of patients with cancer adopted the maladaptive coping strategy “Helpless-Hopeless”, measured on mini-MAC scale.^31^ In an interview study of 42 women diagnosed with advanced breast cancer 4 years previously, women with transient distress adopted various strategies to manage with the demands related to their cancer, including acceptance, social support, and taking an active command over what had happened.^32^ Such strategies have similarities with the “Fighting spirit” strategy that in our study was related to lower occurrence of nausea than in patients adopting maladaptive strategies. Women with ovarian cancer (n = 162) reported the plausible benefit of learning adaptive strategies during emetogenic cancer therapy. Those who learned adaptive strategies, including for example self-care strategies and relaxation training, added to a less effective antiemetic treatment, experienced even better wellbeing than patients who did not receive the strategy-learning intervention but received modern, effective antiemetic treatment.^33^ A randomized controlled study found that adoption of the maladaptive coping strategies “Helpless-Hopeless” and “Anxious Preoccupation” decreased in patients receiving an integrative non-pharmacological therapy during chemotherapy,^34^ which was seen also in a similar intervention study during radiotherapy.^35^ The less “Helpless-Hopeless” and the more “Fighting Spirit,” the better the level of functioning during chemotherapy was reported.^18^ Even during the last year in life, palliative patients experienced better QoL when adopting less “Helpless-Hopeless” coping.^42^ Our and previous findings highlight the importance of cancer care professionals’ support to patients regarding their choice of coping strategies in their burdensome situation,^13,14,29^ potentially doing so after identifying the patient’s coping strategies using the MAC-scale.

We adopted Roy’s Adaptation Model^20,21^ to understand the adjustment to the stressful and burdensome situation during radiotherapy. The adjustment seems to be a process involving perceptual and information processing, learning from previous expectations, and judgment, that is, expectation. The adjustment also involves biological reactions (in the case of nausea, eg, a release of serotonin) and emotions (eg, hopelessness, and anxiety).^3,20,21^ If an individual adopts adaptive coping strategies to cope with the demands,^11,30,32,33^ and has positive expectations,^9,10^ this hypothetically modifies the biological reactions,^15,21,22,30,43,44^ which in turn creates less nausea. Exposure to previous nausea experience increases the risk for new nausea experiences by biopsychosocial adaptation mechanisms.^15^ Expectations are theoretically considered to be judgments based on learning adapted from previous situations.^11^ Interestingly, this was valid also for our study patients in this clinical cancer care setting. The observation that the patients who experienced nausea during the radiotherapy period more often were patients who had expected themselves to become nauseous based on previous nausea experiences, compared to patients who did not experience nausea, is in accord with previous findings during chemotherapy (n = 671). The patients who expected to experience nausea experienced double the nausea of patients not expecting nausea.^45^ Of patients who did not expect themselves to become nauseous when undergoing chemotherapy, 81% did not experience nausea, compared to 68% of those expecting nausea.^36^ Our finding is also in line with observations that experiences of previous nausea in other situations increased the risk for radiotherapy-induced nausea, or for anticipatory nausea,^11,37^ which make it important to identify patients who believe themselves to be at higher risk of nausea than others.

Our study patients’ reasons for expecting nausea during the radiotherapy period most often were that they had previous nausea experiences. This was in line with descriptions from patients during emetogenic chemotherapy, which indicates that previous experiences were even more important than the information received from cancer care professionals in shaping expectations and thus new nausea experiences.^46^ A previous randomized controlled trial found that extended information on what to expect when undergoing emetogenic chemotherapy was seen to mitigate severe nausea. Another randomized controlled study found that cancer care professionals can strengthen the patients’ positive expectations. Patients who before an expectancy-strengthening intervention had expected themselves to become nauseous when undergoing radiotherapy needed to consume less rescue antiemetic medications after receiving the expectancy-strengthening intervention instead of just neutral information.^9^ According to Roy’s Adaptation Model, information processing and learning from experiences are pointed out as being important in the adaptation process.^20,21^ We believe that providing patients with information that creates a realistic expectation of what radiotherapy involves may support the patients to feel prepared to cope with the stressors and demands experienced when undergoing radiotherapy.^38^ A previous study found waiting for radiotherapy was associated with more stress than undergoing the radiotherapy procedures, probably since the patients did not know what to expect during the waiting period.^47^ In the current study, the rationales for expecting nausea during the radiotherapy period were rather similar in patients who felt that they were on “much higher risk” and in patients who felt that they were on “higher risk.” Accordingly, cancer care professionals may consider applying a simple three-graded category scale (“Lower risk,” “Similar risk,” “Higher risk”), to identify patients with higher nausea expectations than others. Cancer care professionals in integrative oncology^12^ may thus consider supporting patients regarding their expectations and coping strategies, especially patients with previous nausea experiences.

A strength of this study is the longitudinal measure of nausea during the entire radiotherapy period,^4^ with high response rates. This allows us to investigate valid relations between the baseline coping measure^5,27^ and rationales for expectations regarding nausea and the true onset of nausea during the radiotherapy period. The study followed the hierarchical step model^48^ to avoid bias in the design and the interpretation of data. Thus, the study included a rather large study population size without selection errors, and identified and measured potential confounding factors providing good precision. However, a weakness of the study is that the data collection did not measure all variables that may influence nausea or coping, such as social support and personality.^23,41,49,50^ The study avoided interviewer-related bias by giving questionnaires to be answered privately in written forms that were returned in secrecy to avoid conscious bias. The response rate of 87% regarding MAC scale was lower than the very high response rates regarding nausea, vomiting and patients’ own estimation of their risk for nausea. However, the MAC scale’s response rate still is high.^51^ Two thirds of the patients responded to all 40 items; these patients were somewhat younger and consisted of more women than the group of patients lacking responses. An individual’s coping capacity may vary with time, with variations in experienced burden,^6,17-19,26,47^ and due to support from others.^13,23,26^ Cancer care professionals sometimes underestimate patients’ burden associated with nausea and, therefore, overestimate the patients’ coping capacity.^31^ Thus, it seemed important for us to assess the patients’ coping capacity by using a self-assessment instrument, that is, the MAC scale.^5,27^ The MAC scale has widely been used previously,^6,19,24,26-28^ including in Swedish samples.^24,27,49^ Previous studies presented that the MAC scale may have different factors with respect to the original factors, particularly when using a version translated from one original language (English) to another language (eg, Swedish).^27^ Thus, the study applied the five-factor model,^5^ according to the factor-analysis for the Swedish version used in the study,^27^ relevant for the target population. The evaluator used statistical methods relevant to the level of data. However, instead of just comparing groups of patients experiencing or not experiencing nausea, future studies may statistically analyze changes, trajectories, and stability of the daily nausea rating over time during the assessment period,^4^ using for example a linear mixed model. It is plausible that the coping strategies might be predictive of dynamic changes rather than of occurrence of nausea or vomiting at least once during the entire radiotherapy period. On the other hand, coping strategies may change over time,^26^ based on contextual factors and the new experiences during the radiotherapy period, shaping modified expectations.^10,11^ Most of the study participants were middle-aged or older women, treated for gynecological cancer and all were in a condition with the mental and physical capacity to give informed consent, receiving antiemetic penetrating or non-penetrating acupuncture added to antiemetics. The generalizability of the study findings to men, to younger patients, to other irradiated fields for other cancer diagnoses, receiving other antiemetic therapies, and to patients in worse condition may accordingly be limited, since coping strategies may be different in men,^40^ depending on age,^23,35^ and cancer diagnosis.^22,49,50^

The study findings indicate that patients adopting maladaptive coping strategies, or who expect nausea based on previous nausea experiences, were more likely to experience nausea than other patients when undergoing emetogenic pelvic-abdominal radiotherapy. Cancer care professionals may identify patients adopting maladaptive coping strategies or having high nausea expectations by applying the MAC scale and self-assessment of expected nausea risk and guide these patients to adopt adaptive coping strategies and strengthen their expectations on successful prevention of nausea. Integrative oncology may welcome future efficacy studies on strengthening patients’ coping strategies and positive treatment expectations in patients undergoing emetogenic cancer therapies.
